# Complete Genome Sequence of *Bacillus cereus Sensu Lato* Bacteriophage Bcp1

**DOI:** 10.1128/genomeA.00334-14

**Published:** 2014-06-12

**Authors:** Raymond Schuch, Adam J. Pelzek, Monica M. Fazzini, Daniel C. Nelson, Vincent A. Fischetti

**Affiliations:** Laboratory of Bacterial Pathogenesis and Immunology, The Rockefeller University, New York, New York, USA

## Abstract

*Bacillus cereus sensu lato* organisms are an ecologically diverse group that includes etiologic agents of food poisoning, periodontal disease, and anthrax. The recently identified Bcp1 bacteriophage infects *B. cereus sensu lato* and is being developed as a therapeutic decontamination agent and diagnostic countermeasure. We announce the complete genome sequence of Bcp1.

## GENOME ANNOUNCEMENT

Rapid detection, decontamination, and therapeutic interventions for *Bacillus cereus sensu lato* group members are sought (1–3), and promising bacteriophage-based methods have been proposed (4–6). The Bcp1 myovirus represents one such bacteriophage. Bcp1 was originally isolated from a landfill soil sample using soft-agar overlays and single-plaque purification. The infectivity of Bcp1 toward a wide variety of *B. cereus sensu lato* isolates was demonstrated, as described (7). Furthermore, the purified Bcp1 lysin PlyB is a potent bacteriolytic enzyme that adopts a glycosyl hydrolase (GH)-25-like fold (8) common among potentially therapeutic cell wall-cleaving enzymes (9). The ability of the Bcp1 phage, and its encoded lysin, to recognize and kill *B. cereus sensu lato* bacteria makes the phage (and its components) attractive as a tool to detect and eradicate these organisms.

Genomic DNA was extracted from Bcp1 using the Lambda Maxi DNA purification kit (Qiagen). DNA sequencing was performed by Macrogen (Seoul, South Korea) using an ABI3700 automatic sequencer (Applied Biosystems) in the manner described (10). Briefly, genomic DNA was sheared using a nebulizer (Invitrogen), blunt-end repaired, and dephosphorylated. DNA fragments in the 1- to 6-kb range were ligated into the pCR4Blunt-TOPO vector (Invitrogen) and electroporated into *Escherichia coli* DH10B cells. Clones were sequenced until >11-fold redundancy was obtained, and data comprised of 2,880 reads and 1,290,542 nucleotides were assembled into three contigs. Unlinked contigs were completed by primer walking (Genewiz). To confirm the ends of the linear Bcp1 genome, DNA was purified from Bcp1-infected *Bacillus anthracis* and primers facing the downstream and upstream ends of the phage DNA were used to amplify genome junctions of concatemeric intermediaries; resulting PCR products were sequenced. The genome was assembled using SeqMan Pro (version 11.2.1; DNASTAR) software. Open reading frames (ORFs) were identified using GeneMark.hmm for prokaryotes (11), SeqBuilder (version 11.2.1, DNASTAR), and ORF Finder (NCBI).

The double-stranded DNA (dsDNA) genome of Bcp1 consisted of 152,778 bp and contained 229 ORFs. The ORFs were divided into modules related to head structure and packaging machinery (including major capsid protein, portal protein, terminase, and prohead protease), baseplate structure, tail structure (tail fibers, tail sheath, and tail-associated protein), phage DNA synthesis (DNA helicases I and II, exonuclease I, DNA primase, and DNA polymerase), and host lysis (lysin and holin). Based on genome size, DNA sequence, gene order, and protein sequences, Bcp1 is most similar to *Bacillus cereus* myoviruses vB_BceM_Bc431v3 and BCP78 (GenBank accession numbers NC_020873 and NC_018860, respectively).

Bcp1 carries several genes predicted to modify bacterial adaptive behaviors. Such loci encode three RNA polymerase sigma factors, two of which drive conversion of *B. anthracis* to asporogeny (12), as well as a metallo-beta-lactamase and a protein, YhbH, required for sporulation in *Bacillus subtilis*. Most importantly, five Bcp1 proteins (including the lysin, tail fibers, and baseplate) with predicted cell wall hydrolase activity were identified; such proteins may serve as decontamination and/or therapeutic agents. The lysin and tail fibers also contain putative *B. cereus sensu lato* surface-binding domains and are thus potential diagnostic and detection reagents.

### Nucleotide sequence accession number.

The GenBank accession number for Bcp1 is KJ451625.
